# When Playing Is a Problem: An Atypical Case of Alien Hand Syndrome in a Professional Pianist

**DOI:** 10.3389/fnhum.2017.00198

**Published:** 2017-04-24

**Authors:** Arantxa Alfaro, Ángela Bernabeu, Francisco J. Badesa, Nicolas García, Eduardo Fernández

**Affiliations:** ^1^Department of Neurology, Hospital Vega Baja de OrihuelaAlicante, Spain; ^2^Bioengineering Institute, University Miguel HernándezElche, Spain; ^3^Centro de Investigación Biomédica en Red en Bioingeniería, Biomateriales y Nanomedicina (CIBER-BBN)Madrid, Spain; ^4^Magnetic Resonance Department, Inscanner S.L.Alicante, Spain; ^5^nBIO Research Group, University Miguel HernándezElche, Spain

**Keywords:** alien hand syndrome, corticobasal syndrome, diffusion tensor imaging (DTI), piano playing, self-body perception

## Abstract

Alien hand syndrome (AHS) is a neurological illness characterized by limb movements which are carried out without being aware of it. Many patients describe these movements as aggressive and some perceive a strong feeling of estrangement and go so far as to deny ownership. The sense of body ownership is the perception that parts of one’s body pertain to oneself, despite it is moving or not and if movement is intentional or unintentional. These anomalous self-experiences may arise in patients with focal brain lesions and provide unique opportunities to disclose the neural components underlying self-body perception. The feeling of foreignness described in AHS is often observed in post-central cortical lesions in the non-dominant hemisphere and is typical of the “posterior alien hand variant”. We used Diffusion-Tensor magnetic resonance imaging (DT-MRI) in an unusual case of posterior AHS of the dominant hand in a professional pianist with corticobasal syndrome (CBS). The patient showed uncontrolled levitation with the right arm while playing the piano and perceived as if her hand had a “mind of its own” which prevented her from playing. MRI-scans show asymmetric brain atrophy, mainly involving left post-central regions and SPECT-Tc99m-ECD patterns of hypometabolism over the left parietal-occipital cortices. DT-MRI revealed extensive damage which comprised left fronto-temporal cortex and extends into the ipsilateral parietal cortex causing a disruption of corpus callosum (CC) projections from the rostrum to the splenium. Our case illustrates that posterior AHS may occur in the dominant hemisphere due to widespread damage, which exceed parietal cortex. The parietal lobe has been recognized as a multimodal association region that gets input from several networks and organizes motor output. We suggest that the disturbance to this pathway could result in disruption of motor output and associate an abnormal motor control and anomalous self-body perception.

## Introduction

Alien hand syndrome (AHS) is one of the most gripping disconnection disorders in neurology. It could be described as the perception that one limb “has its own volition” together with recognizable uncontrolled motor activity which pries with the voluntary movements of the unaffected limb (Doody and Jankovic, 1992). It is fairly common that the affected arm holds clothes, parts of the body, adjacent objects or even people (Josephs and Rossor, 2004). Moreover, patients are usually unaware of it, and could display signs of inattention of the affected limb, perceiving that it is not theirs (Josephs and Rossor, 2004).

AHS is observed in post-stroke patients, secondary to vascular malformations and brain tumors, neurosurgical lesions, trauma and neurodegenerative diseases, particularly in atypical parkinsonian syndromes as corticobasal syndrome (CBS) and progressive supranuclear palsy (Scepkowski and Cronin-Golomb, 2003; Chang et al., 2012; Alexander et al., 2014). The presence of limb apraxia, visuospatial dysfunction and AHS is suggestive of CBS, particularly when it develops in a progressive way. In fact, alien hand phenomenon appears in around 30% of compiled CBS cases (Armstrong et al., 2013). Neural mechanisms of AHS have remained speculative and the combination of lesions necessary to produce this phenomenon is uncertain (Scepkowski and Cronin-Golomb, 2003). According to the anatomical lesions and clinical features, three different categories: callosal, frontal and posterior AHS have been described. The first two types are classified as an anterior form of AHS whereas the third one is also defined as a posterior form (Scepkowski and Cronin-Golomb, 2003). The more common “anterior or motor” AHS is characterized by uncontrollable manipulation of objects and involuntary grasping of the dominant hand. Posterior subtype (pAHS) is uncommon and usually associated with involuntary movements such as a position-dependent levitation of the arm in addition to a sensation of strangeness in the limb (Scepkowski and Cronin-Golomb, 2003). The etiology of involuntary movements in pAHS is not elucidated yet and remains unclear (Armstrong et al., 2013). It mostly, though not exclusively, affects the non-dominant hand with lesions involving the posterior right hemisphere (Kessler and Hathout, 2009). Shared mechanisms between AHS variants have been described and the data seem to indicate that most cases of AHS arise from lesions of interhemispheric networks or between the frontal and the parietal lobes (Sarva et al., 2014). However, the case-report descriptions of patients with damage distant from the typical affected areas reflects our partial knowledge of the processes producing AHS (Sarva et al., 2014).

We describe a case of pAHS of the dominant right hand secondary to CBS in a 65-year-old professional pianist with unusually increased alien limb symptoms while playing. Diffusion-Tensor magnetic resonance imaging (DT-MRI) and fiber tractography could offer the opportunity to shed light on the pathophysiology of AHS and other neurological disorders affecting the perception of one’s own body.

## Case Report

### Patient History

A 65-year-old woman, right-handed professional pianist suffered from increasing awkwardness of her dominant arm during the last 5 years. She was healthy until the age of 60, when she first felt impairment of the voluntary movement of her right hand while playing the piano. She experienced as whether her arm “didn’t do what it was ought to” and declined to play due to it was “too clumsy to practice”. Rarely, when she moved her left hand, the right one raised involuntarily. She felt strange and surprise with the behavior of her affected arm and believed that “it had an entity of its own”. After 2 years, she had severe difficulties with playing and, although her right hand was not paretic, her movement was significantly slowed down. The hand carried on its odd compartment, which utterly hampered her from playing. No history of any other illnesses, toxins or drugs were reported. The patient underwent a detailed assessment by a neurology specialist. Its main features on clinical examination were asymmetric hand clumsiness, rigidity and bradykinesia with reduced right arm swing, prominent right constructional and ideomotor apraxia and feelings of estrangement of the right limb coupled with non-purposeful movements such as levitation, especially when attention decreased well distinguishable from distal pseudo-athetosis which was not presented. She exhibited other cortical sensory deficits such as decreased pain sensation in the right side besides transcortical motor aphasia. Clinical criteria for dementia were absent.

The patient was diagnosed as having probable CBS based on recently published criteria (Armstrong et al., 2013; Alexander et al., 2014) furthermore, she displayed the typical features of pAHS. She was treated with levodopa (until 800 mg per day) and clonazepam (1 mg per day). However, she had modest response to it.

### Methods

As part of the clinical assessment, MRI was performed in a 3T MR scanner (Philips Achieva, Philips Medical Systems, Nederlands) with a SENSE Neurovascular coil (16 elements). No contrast agent or sedation was utilized. For the MRI protocol a high-resolution T1-weighted gradient-echo scan: 212 slices, 0.8 mm isotropic voxels, FOV 250 × 250 mm, TR 11 ms and TE 4.9 ms was acquired. DT Imaging (DTI) was acquired in axial slice orientation, using a single-shot EPI sequence with diffusion encoding in 32 directions (values 0 and 800 s/mm^2^, voxel size was 2 × 2 × 2 mm^3^, 60 slices, SENSE factor 1.9). The diffusion-weighted data were transferred to a workstation for analysis and eddy current compensation was performed by affine registration to B0 image. Tractography was carried out using the PRIDE fiber-tracking tool (Philips Medical Systems) as described previously (Alfaro et al., 2015; Bernabeu et al., 2016) and was fulfilled based on the connection between two areas of regions of interest (ROIs), the ROIs were drawn manually based on the anatomical MRI and on published atlases (Wakana et al., 2004). The fibers were computed automatically by the software with the following parameters as stopping criteria: minimum fractional anisotropy value (FA) of 0.3, maximum fiber angle between fibers of 27° and minimum fiber length of 10 mm.

Additionally, brain perfusion studies with SPECT-Tc99m-ECD were performed with a Philips Forte Gamma Camera System (Philips Medical Systems, US) using Tc-99m radiopharmaceuticals. Imaging acquisition and reconstruction was carried out with the usual specified protocols (Delrieu et al., 2010).

The study adhered to the Declaration of Helsinki. The protocol was approved by the institutional review board (Hospital Vega Baja Ethics Committee). The patient gave her written informed consent before entering the study and for publishing the information appearing in this case report.

### Results

**Figure 1:**
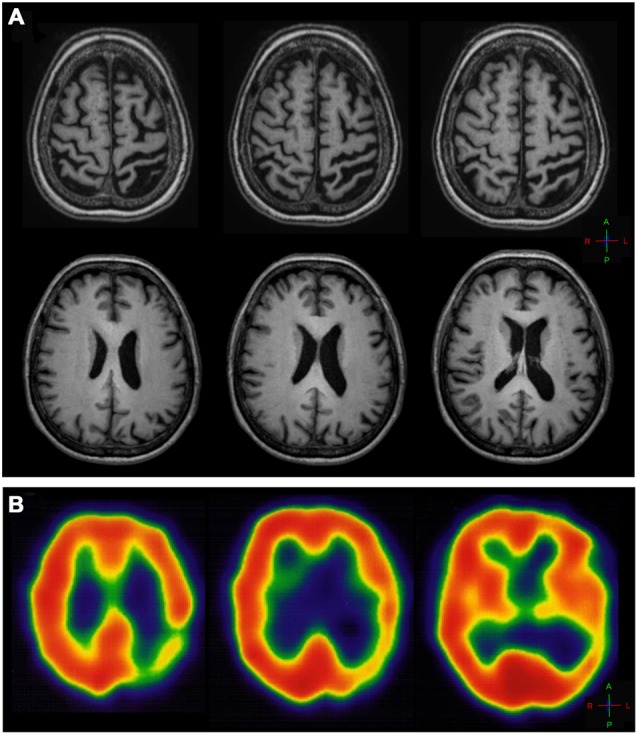
**(A)** T1-weighted axial MR image of the brain showing diffuse cortical atrophy and ventriculomegaly in the left hemisphere, marked in the left central sulcus and precentral gyrus and more prominent on the left post-central gyrus and parietal-posterior area. **(B)** SPECT axial slices of thechnetium-99m-HMPO brain perfusion scan showing deficient cerebral perfusion in the left posterior parietal-occipital cortex.

**Figure 2:**
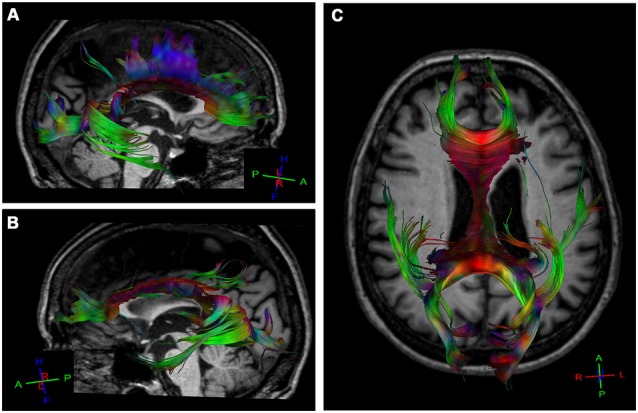
**Diffusion tensor tractography (DTT) for corpus callosum (CC) fibers using a sensitive-encoding head coil on a 3.0T Philips Achieva system.** DTT was performed based on the connection between two regions of interest (ROI) in order to minimize the risk of including other tracks. **(A)** Right CC fibers extended normally to frontal, temporal, parietal and occipital cortices. **(B)** Extensive disruption of the left CC connections from the rostral body to the splenium. A small group of CC fibers in both brain hemispheres are preserved. **(C)** Axial reconstructed of CC fibers in the patient.

MR imaging of the brain revealed severe atrophy in the left hemisphere, mainly in the left posterior post-central gyrus, anterior and posterior parietal lobe and ipsilateral occipital cortex (Figure 1A) consistent with the SPECT-Tc99m-ECD result, which displayed deficient cerebral perfusion in all these regions (Figure 1B). DTI-MRI showed right corpus callosum (CC) fibers connected properly to frontal, temporal, parietal and occipital cortex (Figure 2A). By contrast, left CC fibers displayed serious and wide disruption, which affected left premotor, supplementary motor and motor cortex connections besides left temporal, parietal and occipital cortices connections further extensive damage in the left superior longitudinal fascicule (Figure 2B). A little group of CC fibers in both brain hemispheres, crossing through the rostrum and the genu, were preserved (Figure 2C).

## Background

Studying the abnormalities of self-body perception due to brain damage has a key role in addressing questions regarding the structure and functional signature of body consciousness (Pia et al., 2013). This is the case of patients with pAHS who commonly manifest body schema distortions such as the strong feeling of foreignness or strangeness of one limb and other parietal sensory deficits (Doody and Jankovic, 1992; Scepkowski and Cronin-Golomb, 2003; Josephs and Rossor, 2004).

In the last 20 years our understanding of AHS subtypes has evolved to the three, well-defined variants: the two anterior (frontal and callosal) variants and the relatively recent added posterior one (Sarva et al., 2014). The “posterior form” of AHS has been related with impairment to the thalamus, the posterolateral parietal cortex and the occipital lobe (Scepkowski and Cronin-Golomb, 2003; Prakash et al., 2011). This variant usually, though not exclusively, involves the non-dominant limb (Kessler and Hathout, 2009; Kloesel et al., 2010). The alien limb movements appear non-purposeful and non-conflictual and patients could experience involuntary levitation of the arm which may be task specific (Rohde et al., 2002; Gondim et al., 2005; Prakash et al., 2011). In some cases, the alien hand also could exhibit a bizarre position, called “parietal hand” in which the palmar surface is withdrawn from approaching tools and the fingers move into an extremely extended posture (Prakash et al., 2011; Sarva et al., 2014). Additionally, pAHS can be accompanied by hemianesthesia, hemianopia, visuospatial neglect (Yuan et al., 2011) and optic ataxia (Levine and Rinn, 1986) and some patients may have significant sensory deficits without weakness (Spector et al., 2009).

pAHS could be produced by different neurodegenerative conditions for instance, Creutzfeld–Jacob disease (Rubin et al., 2012), Alzheimer’s disease, CBS or progressive supranuclear palsy (Chand et al., 2006) as well as cerebrovascular accidents in the thalamus, parietal cortex or posterior cerebral artery (Marey-Lopez et al., 2002; Rohde et al., 2002; Gondim et al., 2005; Hassan and Josephs, 2016).

Due to the relative low prevalence of this syndrome and the limited reports of pAHS described, our understanding of underlying mechanisms remains incomplete. On the one hand, some authors noted the necessary implication of parietal lobe in pathophysiology of pAHS. The parietal lobe is a multimodal association area required for formation of proprioceptive schemes which assist in the integration of body image (Perez-Velazquez, 2012). Additionally, it receives inputs from primary somatosensory and prefrontal cortices and coordinates motor output (Perez-Velazquez, 2012). Because of this, damage to parietal lobe could produce inability to combine sensory input and motor output and may induce impaired volitional movement execution, involuntary arm levitation and release a pronounced feeling of estrangement of a limb (Graff-Radford et al., 2013). On the other hand, a distortion of body representation due to an anomalous cortico-striato-thalamic network without significant parietal lobe injury has been recently described as a cause of pAHS (Filevich et al., 2012). Moreover, it is known that a thalamic stroke with no frontal and parietal involvement may result in pAHS with slight sensory loss (Bartolo et al., 2011). Likewise, posterior cerebral artery stroke may evoke sensation of limb foreignness secondary to the damage to the medial paralimbic fibers implicated in limb awareness (Groom et al., 1999). According to this, we could say that nowadays, the neuroanatomical circuitry involved in pAHS is diverse and yet poorly understood.

## Discussion

Here we focused on an unusual patient affected by CBS who exhibited a constellation of symptoms consistent with pAHS in her dominant right limb. Nevertheless, our case differs from other reported cases of pAHS.

First of all, pAHS is classically described in non-dominant limb (Scepkowski and Cronin-Golomb, 2003) and there have been very few reports of lesions in the left hemisphere causing pAHS of the dominant right upper extremity (Carrilho et al., 2001; Rohde et al., 2002; Kessler and Hathout, 2009; Kloesel et al., 2010). Leiguarda et al. (1993) described a patient who developed right AHS following neurosurgical removal of a vascular malformation from the left parietal cortex and Gondim et al. (2005) reported a position-dependent levitation of the dominant limb afterward left parietal cerebrovascular accident. Nevertheless, there is anecdotal evidence from dominant pAHS with atrophy in the left dominant parietal lobe. Kessler and Hathout (2009) propose a precise localization of AHS of the dominant hand through the report of a patient with left parietal stroke and suggests that Brodmann area 5, which coincides with the tertiary somatosensory cortex and is required in stereognosis and post-central circumvolution, which is entailed in kinesthesia, could trigger the anomalous movements and the sensations of strangeness of an alien arm, even in the dominant limb (Sarva et al., 2014). In the light of these observations and our reported findings, it seems that pAHS variant could not be restricted to non-dominant hemispheric lesions.

Otherwise, although clinically our patient presented symptoms that remind one of the posterior alien hand variant, neuroimaging revealed extensive damage that exceeds the posterior parietal cortex causing a widespread disruption of left CC connections from the rostral body to the splenium. These results suggest that the sense of ownership over the alien hand could be established by a wide spectrum of lesions, ranging from purely anterior to purely posterior forms and hinted that disruption of the motor centers from the parietal cortex probably cause misperception, and developing an abnormal integration between afferent multisensory signals and pre-existing body presentations and the loss of consciousness of movement (Graff-Radford et al., 2013; Sarva et al., 2014).

Furthermore, our patient exhibited a previously unreported feature: apparently her posterior alien arm symptoms exacerbated while piano playing. Playing the piano requires the activation of multisensory and motor networks located in distant but functionally related brain regions such as frontal, parietal, and temporo-occipital cortices besides subcortical structures such as basal ganglia, thalamus and cerebellum (Altenmüller and Schlaug, 2015). Indeed, parietal lobe and temporo-occipital cortices play a critical role for conscious perception of sensory information. These areas work together in order to integrate inputs from the auditory, visual, and somatosensory system into a combined sensory impression (Altenmüller and Schlaug, 2015). The functional links between all these brain regions make possible the coupling of perception and action for playing. As we described before, our patient suffered from a widespread disruption of brain networks involved frontal, parietal and temporo-occipital cortices and first perceived impairment of the conscious and voluntary movements of her right arm while playing the piano. It has been known that uncoordinated hand movements or involuntary levitation in patients with pAHS may be task-specific. Kloesel et al. (2010) described a patient with pAHS secondary to CBS who had exaggerated arm elevation only while walking (Prakash et al., 2011) and in other cases, involuntary movements are triggered or worsened by tactile stimulation (Gondim et al., 2005), sudden noises or coughing (Rohde et al., 2002). Levitation in our patient appeared when attention decreased and were not related to a specific position of the arm. It is certain that levitation exacerbated while piano playing. In addition, she first noticed impairment of the controlled movement of her right hand during piano execution. However, we consider that playing a musical instrument demands the suitable perception of the limb position and motion in space and requires a fine visual, proprioceptive and motor integration (Pascual-Leone, 2001). Most of the networks which take part in these processes are damaged in our patient.

Regarding the differential aspects of our findings with task-specific dystonia in pianists, our patient did not display the typical cramps, hyperextensions and flexions of the hand while playing the piano which is commonly described in task-specific dystonia of musicians. It is known that the posterior variant of AHS can be accompanied by other features such as atypical hand posture sometimes referred to as a “parietal hand” (Prakash et al., 2011) and other cortical sensory deficits like hemianesthesia and hemineglect which cause a poor proprioceptive awareness and could restrict the skill for playing (Scepkowski and Cronin-Golomb, 2003).

## Concluding Remarks

This case report shows that pAHS could appear in the dominant limb from a widespread disruption of brain networks which exceeds left posterolateral parietal and occipital cortices. Moreover, these symptoms could get worse during a specific task as playing the piano. Further imaging research is needed in order to understand the neural pathways involved in pAHS. A combination of neurological assessment and anatomical and functional imaging may provide invaluable information about relationship between clinical features and anatomic localization of pAHS and contribute to further expand our knowledge about this rare condition and anomalous self-body perception.

## Author Contributions

AA, AB and EF designed the study. AA was responsible for clinical data on the case. AB contributed to the acquisition and analysis of images. AA, EF, AB, FJB and NG carried out the interpretation of data. AA and EF prepared the final version of the article.

## Funding

This work has been partially supported by Spanish grant MAT2015-699767-C3-1-R and by CIBER BBN.

## Conflict of Interest Statement

The authors declare that the research was conducted in the absence of any commercial or financial relationships that could be construed as a potential conflict of interest.
